# Prediction of microRNAs affecting the syncytin-1 (HERV-W) and syncytin-2 (HERV-FRD) genes regulation in endometriosis and miscarriage

**DOI:** 10.22099/mbrc.2025.54457.2225

**Published:** 2026

**Authors:** Mehdi Gholami-Barzoki, Somayeh Shatizadeh-Malekshahi, Mohammad Shayestehpour, Haleh Soltanghoraee

**Affiliations:** 1 Department of Virology, Faculty of Medical Sciences, Tarbiat Modares University, Tehran, Iran; 2 Department of Bacteriology and Virology, Faculty of Medicine, Isfahan University of Medical Sciences, Isfahan, Iran; 3 Reproductive Biotechnology Research Center, Avicenna Research Institute, ACECR, Tehran, Iran

**Keywords:** microRNA, Endometriosis, Miscarriage, Human endogenous retroviruses

## Abstract

Abnormal expression levels of microRNAs are associated with numerous diseases in the female reproductive tract. A small subset of human endogenous retroviruses (HERVs) genes have retained open reading frames (ORFs) that serve beneficial functions for the host. Syncytin-1 (HERV-W) and Syncytin-2 (HERV-FRD) play crucial roles in mammalian development and are expressed in placental trophoblasts. The miRNAs associated with HERV-W and HERV-FRD in spontaneous abortion and endometriosis have not been elucidated. The present study aimed to identify potential miRNAs that affect the regulation of Syncytin-1 and Syncytin-2 in endometriosis and miscarriage using bioinformatics tools. Complete CDS of Syncytin-1 (ERVW-1) and Syncytin-2 (ERVFRD-1) genes were collected from the gene bank database. Several target prediction algorithms were utilized, such as TargetScan, DIANA, miRDB, and miRWalk. Complete CDS of Syncytin-1 (ERVW-1) and Syncytin-2 (ERVFRD-1) genes were collected from the gene bank database. By integrating data from these diverse bioinformatics databases, miR-509-3p and miR-625-5p were consistent across multiple platforms, ensuring robust selection criteria. These tools facilitate the identification of differentially expressed miRNAs, understanding their roles in cellular processes, and potentially utilizing them as biomarkers for disease diagnosis and prognosis. Validation of the identified miRNAs in experimental models or clinical samples is needed to confirm their roles in endometriosis and miscarriage.

## INTRODUCTION

Bioinformatics is a multidisciplinary field and has become essential in modern molecular biology research. In studies investigating gene regulation, bioinformatics enables the efficient prediction and prioritization of candidate molecular targets, such as microRNAs, facilitating the understanding of disease mechanisms and accelerating the development of diagnostic biomarkers and novel therapies [1, 2]. MicroRNAs (miRNAs) constitute a class of short, non-coding, single-stranded RNA molecules approximately 18–22 nucleotides in length. These molecules serve as critical regulators of gene expression by promoting mRNA degradation or translational repression at the post-transcriptional level [3]. It is estimated that miRNAs influence up to 60% of protein-coding genes within the human genome by modulating translation [4]. Aberrant miRNA expression has been associated with multiple pathological conditions [3]. 

The formation of miRNAs involves a series of sequential steps that take place across both a nuclear and a cytoplasmic phase. They are generally transcribed by RNA polymerase II or III as elongated primary transcripts known as primary miRNAs (pri-miRNAs), which are subsequently cleaved to generate precursor miRNAs (pre-miRNAs). These pre-miRNAs undergo additional processing to produce miRNA duplexes. The resulting duplexes are then incorporated into Argonaute (AGO) proteins through the action of the RNA-induced silencing complex (RISC), a complex composed of Dicer, trans-activation response RNA-binding protein (TRBP), and AGO. TRBP functions as an essential factor in distinguishing between the "guide" and "passenger" strands within the miRNA duplex by detecting differences in their thermodynamic stability. It identifies the strand with the less stable 5′ end and accordingly assists in its proper orientation and loading onto AGO proteins. Afterward, AGO unwinds the duplex, removes the passenger strand, and retains the mature miRNA that carries out gene regulation [5]. 

Abnormal expression levels of miRNAs are associated with numerous female reproductive tract diseases, including preeclampsia, uterine leiomyoma, ovarian adenocarcinoma, endometriosis, and recurrent spontaneous abortion [6]. Endometriosis is a medical condition distinguished by the growth of endometrial tissue beyond the uterus, affecting millions of women worldwide, and can lead to chronic pelvic pain, infertility, and other debilitating symptoms [7]. In endometriosis, aberrant expression of miRNAs has been observed in the ectopic endometrial tissue. These altered miRNAs can influence gene expression patterns, leading to abnormal cell proliferation, migration, and invasion [8]. Miscarriage is the spontaneous loss of a pregnancy before the 20th week. It is often caused by chromosomal abnormalities in the fetus, maternal health conditions such as uterine or cervical issues, hormonal problems, thyroid disease, obesity, infections, etc [9]. The dysregulation of miRNAs has been linked to recurrent miscarriage, and their profiling is considered a promising area for the development of early diagnostic biomarkers and preventive care. 

Human endogenous retroviruses (HERVs) are remnants of ancient retroviral infections and now constitute about 8% of the human genome. Although most HERV sequences have accumulated mutations, deletions, or rearrangements over evolutionary time, a limited number have maintained open reading frames (ORFs) that provide advantageous functions to the host. Among these functional elements, Syncytin-1 (derived from HERV-W) and Syncytin-2 (originating from HERV-FRD) are particularly significant. Through their fusogenic ability, Syncytin-1 and Syncytin-2 actively contribute to the process of placenta development by facilitating the fusion of cytotrophoblasts, ultimately forming the syncytiotrophoblast layer [10]. 

Trophoblast fusion is tightly regulated by physiological processes, including decidualization, epithelial-mesenchymal transition (EMT), and local inflammatory signaling, all of which modulate Syncytin expression and activity [11]. Decidualization, the hormonally driven transformation of endometrial stromal cells, influences trophoblast invasion and fusion capacity, while EMT contributes to cellular plasticity and invasiveness in endometriotic lesions [12]. Dysregulation of these regulatory axes likely impairs Syncytin-mediated fusion, contributing to pathologies such as miscarriage, intrauterine growth restriction, preeclampsia, and endometriosis [13].

While miRNA expression patterns have been described in many embryonic, physiological, and oncogenic processes, the miRNAs associated with HERV-W and HERV-FRD in spontaneous abortion and endometriosis have not been elucidated. The present study aimed to design potential miRNAs that affect the regulation of Syncytin-1 (HERV-W) and Syncytin-2 (HERV-FRD) in endometriosis and miscarriage by bioinformatics tools. 

## MATERIALS AND METHODS

### Sequence Retrieval:

The complete coding sequences (CDS) of Syncytin-1 (ERVW-1) and Syncytin-2 (ERVFRD-1) genes were collected from the GenBank database available at the National Center for Biotechnology Information Nucleotide database (http://www.ncbi.nlm.nih. gov/genbank/). GenBank is the NIH genetic sequence database and a comprehensive public repository containing annotated DNA sequences submitted from laboratories worldwide. The gene bank contained 50 sequences for ERVW-1 and 53 for ERVFRD-1. The accession numbers of ERVW-1 and ERVFRD-1 complete CDS were NM_001130925.2 and NM_207582.3, respectively. Sequence data were extracted directly from the GenBank ‘Nucleotide’ database to ensure accuracy. Both genes were analyzed for their reference sequence length and transcript variants. The Syncytin-1 CDS is 1,616 base pairs (bp) long, and Syncytin-2 CDS is 1,616 bp. In the NCBI database, ERVW-1 has 2 annotated transcript sequences, and ERVFRD-1 has 2 sequences, inclusive of known isoforms and alternative splice variants. 

### MicroRNAs Prediction Using Bioinformatics Tools:

To predict microRNAs affecting the Syncytin-1 (HERV-W) and Syncytin-2 (HERV-FRD) genes, several target prediction algorithms were employed. TargetScan (Release 8.0, accessed July 10, 2025; https://www. targetscan.org) is a widely used web-based tool that predicts miRNA binding sites based on sequence complementarity, probability of conserved targeting (P_CT), and context++ scoring to infer targeting efficacy. Candidate miRNAs with context++ scores ≤ -0.1 and P_CT ≥ 0.50 were retained. DIANA microT-CDS v5.0 (accessed July 15, 2025; https://dianalab.ece.uth.gr/ microt_webserver/#/) is an advanced platform that predicts miRNA binding sites in both coding sequences and 3′ UTR regions, improving sensitivity by incorporating site accessibility and conservation metrics: MiRNAs with a microT score (miTG) ≥ 0.70 were selected to prioritize high-confidence interactions. miRDB (version updated May 2025; http://mirdb.org/ miRDB/) utilizes a machine learning-based algorithm integrating experimentally validated interactions to predict miRNA targets with specificity scores: MiRNAs with a target prediction score ≥ 80 were included to ensure specificity. miRWalk 3.0 (accessed July 18, 2025; http:// mirwalk.umm.uni-heidelberg.de/) provides a comprehensive resource that searches complete gene transcripts, including 5′ UTR, CDS, and 3′ UTR regions, combining predicted and experimentally validated miRNA-target pairs: Both validated and predicted interactions were considered; however, preference was given to experimentally validated targets when available, otherwise predicted targets with binding scores ≥ 0.70 were included. 

Selection criteria for candidate miRNAs included sequence complementarity, thermodynamic stability, evolutionary conservation, and site accessibility. Results from the individual algorithms were consolidated to generate a list of candidate miRNAs. For further analysis, a scoring table was constructed based on miRNAs predicted by more than two tools. The final list of candidate miRNAs was generated by integrating results across all tools using a weighted voting system. Each miRNA was assigned points based on the number of tools predicting it, the conservation scores, and the presence of canonical seed matches (8-mer > 7-mer > 6-mer). MiRNAs predicted by three or more tools with favorable conservation and site context received the highest priority. In cases of ties, miRNAs with validated targets or stronger context++ scores from TargetScan were favored. This consensus approach ensured rigorous and biologically meaningful candidate selection.

## RESULTS

TargetScan (Release 8: September 2021) is a web-based tool that enables the user to perform searches based on miRNA name, gene name, or miRNA families with different conservation levels across several species. The generated output screen provides rankings of predicted targets based on two criteria: the predicted efficacy of targeting (context+ scores) and the probability of conserved targeting (PCT). The conservation analysis involves evaluating the conservation status of the 3′ UTR region, followed by the analysis of a specific k-mer (8mer, 7mer-m8, or 7mer-1A). The gene’s 3' UTR, which contains the conserved seed sequence, is linked for reference. The context+ score represents the probability of a given target being effectively targeted. This score takes into account various factors, including 3′ compensatory pairing, local AU content, and position contribution (Ref: Common features of microRNA target prediction tools). ERVW-1 was predicted to be targeted by 140 miRNAs, and ERVFRD-1 is predicted to be targeted by 493 miRNAs (Tables 1 & 2). 

**Table 1:** Some microRNAs targeting the ERVW-1 predicted by TargetScan

**Target Rank**	**miRNA Name**	**Position**	**Site type**	**Score**
**1**	hsa-miR-6838-3p	66-73 of ERVW-1 3' UTR	8mer	99
**2**	hsa-miR-5586-3p	360-367 of ERVW-1 3' UTR	8mer	99
**3**	hsa-miR-4667-3p	257-264 of ERVW-1 3' UTR	8mer	99
**4**	hsa-miR-6893-3p	253-260 of ERVW-1 3' UTR	8mer	99
**5**	hsa-miR-370-3p	253-260 of ERVW-1 3' UTR	8mer	99
**6**	hsa-miR-4742-5p	240-247 of ERVW-1 3' UTR	8mer	99
**7**	hsa-miR-629-5p	279-286 of ERVW-1 3' UTR	8mer	98
**8**	hsa-miR-1470	258-264 of ERVW-1 3' UTR	7mer-A1	98
**9**	hsa-miR-3934-5p	164-171 of ERVW-1 3' UTR	8mer	98
**10**	hsa-miR-495-5p	152-159 of ERVW-1 3' UTR	8mer	98
**11**	hsa-miR-7977	112-119 of ERVW-1 3' UTR	8mer	98
**12**	hsa-miR-5699-3p	63-70 of ERVW-1 3' UTR	8mer	98
**13**	hsa-miR-509-3p	171-178 of ERVW-1 3' UTR	8mer	97
**14**	hsa-miR-4684-3p	375-381 of ERVW-1 3' UTR	7mer-m8	97
**15**	hsa-miR-6839-3p	280-287 of ERVW-1 3' UTR	8mer	97

**Table 2:** Some miRNAs targeting the ERVFRD-1 predicted by TargetScan

**Target Rank**	**miRNA Name**	**Position**	**Site type**	**Score**
**1**	hsa-miR-100-3p	1004-1011 of ERVFRD-1 3' UTR	8mer	99
**2**	hsa-miR-6165	817-824 of ERVFRD-1 3' UTR	8mer	99
**3**	hsa-miR-8055	1179-1185 of ERVFRD-1 3' UTR	7mer-m8	98
**4**	hsa-miR-4696	1077-1083 of ERVFRD-1 3' UTR	7mer-m8	98
**5**	hsa-miR-876-3p	1022-1029 of ERVFRD-1 3' UTR	8mer	98
**6**	hsa-miR-656-5p	310-317 of ERVFRD-1 3' UTR	8mer	97
**7**	hsa-miR-6799-3p	274-281 of ERVFRD-1 3' UTR	8mer	97
**8**	hsa-miR-134-3p	136-143 of ERVFRD-1 3' UTR	8mer	97
**9**	hsa-miR-6878-3p	126-133 of ERVFRD-1 3' UTR	8mer	97
**10**	hsa-miR-634	931-938 of ERVFRD-1 3' UTR	8mer	96
**11**	hsa-miR-4722-5p	816-822 of ERVFRD-1 3' UTR	7mer-m8	96
**12**	hsa-miR-4433a-3p	815-821 of ERVFRD-1 3' UTR	7mer-m8	96
**13**	hsa-miR-193b-5p	710-717 of ERVFRD-1 3' UTR	8mer	96
**14**	hsa-miR-4279	672-679 of ERVFRD-1 3' UTR	8mer	95
**15**	hsa-miR-5193	669-676 of ERVFRD-1 3' UTR	8mer	95
**16**	hsa-miR-516a-3p	170-177 of ERVFRD-1 3' UTR	8mer	94
**17**	hsa-miR-625-5p	1107-1113 of ERVFRD-1 3' UTR	7mer-m8	93
**18**	hsa-miR-6758-5p	1190-1196 of ERVFRD-1 3' UTR	7mer-A1	93
**19**	hsa-miR-6794-5p	1108-1114 of ERVFRD-1 3' UTR	7mer-m8	93

In this bioinformatics tool, the prediction scores range from 0 to 100, with candidate transcripts having scores ≥ 50 presented as predicted miRNA targets. This step was performed for every gene separately. ERVW-1 was predicted to be targeted by 27 miRNAs in miRDB. ERVFRD-1 is predicted to be targeted by 46 miRNAs in miRDB (Tables 3 & 4). 

This bioinformatics tool is an advanced miRNA target prediction algorithm that predicts miRNA binding sites in both the 3' Untranslated Region (3'-UTR) and the coding sequence (CDS) with improved performance. The DIANA microRNA prediction tool has a prediction score range from 0 to 100, with candidate transcripts having scores ≥ 50 presented as predicted miRNA targets. Each of the genes ERVW-1 and ERVFRD-1 was predicted to be targeted by 60 miRNAs in the DIANA tool (Tables S1 & S2 Supplementary file). The DIANA microT-CDS server uses a scoring system called "miTG score" to evaluate the predicted interaction strength between miRNAs and their target genes. In Tables S1 and S2, each predicted miRNA is listed alongside its miTG score, which ranges from 0 (indicating an unlikely true interaction) to 1 (signifying a robust prediction, nearly maximum confidence). Only miRNAs above the selected threshold (e.g., miTG ≥ 0.70 or 0.80) were included. 

**Table 3:** List of predicted miRNAs by miRDB targeting ERVW-1

**Target Rank**	**Target Score**	**miRNA Name**	**Gene Symbol**	**Gene Description**
**1**	87	hsa-miR-4774-3p	ERVW-1	endogenous retrovirus group W member 1, envelope
**2**	82	hsa-miR-5586-3p	ERVW-1	endogenous retrovirus group W member 1, envelope
**3**	80	hsa-miR-509-3p	ERVW-1	endogenous retrovirus group W member 1, envelope
**4**	73	hsa-miR-7977	ERVW-1	endogenous retrovirus group W member 1, envelope
**5**	72	hsa-miR-5699-3p	ERVW-1	endogenous retrovirus group W member 1, envelope
**6**	71	hsa-miR-6825-5p	ERVW-1	endogenous retrovirus group W member 1, envelope
**7**	68	hsa-miR-302e	ERVW-1	endogenous retrovirus group W member 1, envelope
**8**	68	hsa-miR-4742-5p	ERVW-1	endogenous retrovirus group W member 1, envelope
**9**	67	hsa-miR-6839-3p	ERVW-1	endogenous retrovirus group W member 1, envelope
**10**	66	hsa-miR-610	ERVW-1	endogenous retrovirus group W member 1, envelope

**Table 4:** List of predicted miRNAs by miRDB targeting ERVFRD-1

**Target Rank**	**Target Score**	**miRNA Name**	**Gene Symbol**	**Gene Description**
**1**	93	hsa-miR-12127	ERVFRD-1	endogenous retrovirus group FRD member 1, envelope
**2**	89	hsa-miR-4696	ERVFRD-1	endogenous retrovirus group FRD member 1, envelope
**3**	86	hsa-miR-3152-5p	ERVFRD-1	endogenous retrovirus group FRD member 1, envelope
**4**	85	hsa-miR-1298-3p	ERVFRD-1	endogenous retrovirus group FRD member 1, envelope
**5**	84	hsa-miR-6794-5p	ERVFRD-1	endogenous retrovirus group FRD member 1, envelope
**6**	84	hsa-miR-625-5p	ERVFRD-1	endogenous retrovirus group FRD member 1, envelope
**7**	84	hsa-miR-4716-3p	ERVFRD-1	endogenous retrovirus group FRD member 1, envelope
**8**	82	hsa-miR-1226-3p	ERVFRD-1	endogenous retrovirus group FRD member 1, envelope
**9**	82	hsa-miR-1303	ERVFRD-1	endogenous retrovirus group FRD member 1, envelope
**10**	75	hsa-miR-204-3p	ERVFRD-1	endogenous retrovirus group FRD member 1, envelope

The miRWalk is a miRNA target site prediction searching the complete transcript sequence, including the 5’-UTR, CDS, and 3’-UTR. miRWalk encompasses both the predicted and experimentally validated miRNA-target interaction pairs. ERVW-1 and ERVFRD-1 were predicted to be targeted by hundreds of miRNAs in miRWalk (Tables S3 & S4 Supplementary file). The microRNAs with the highest score and common among different bioinformatics databases were considered as selected miRNAs. By integrating data from these diverse bioinformatics databases, miR-509-3p and miR-625-5p were consistent across multiple platforms, ensuring robust selection criteria.

## DISCUSSION

Computational approaches are increasingly utilized to identify potential regulatory interactions. Integrating data from multiple prediction algorithms ensures a robust selection of candidate miRNAs, which corresponds with the best practices in bioinformatics analysis. This study emphasizes the importance of understanding the roles of miRNAs in endometriosis and miscarriage. This study aimed to identify miRNAs affecting Syncytin-1 (HERV-W) and Syncytin-2 (HERV-FRD) regulation in endometriosis and miscarriage using bioinformatics tools. miR-509-3p and miR-625-5p were identified as potential key miRNAs across multiple platforms. The identified miRNAs could serve as valuable targets for further research and potentially as diagnostic or therapeutic interventions in the future. Validation of the identified miRNAs in experimental models or clinical samples is needed to confirm their roles in endometriosis and miscarriage. To the best of our knowledge, no studies have yet been conducted investigating the potential miRNAs affecting HERV-FRD and HERV-W gene expression and the possible relationship between them in the samples of spontaneous abortion and endometriosis.

The literature on endometriosis highlights various miRNAs involved in different processes like Epithelial-Mesenchymal Transition (EMT), angiogenesis, cell proliferation, adhesion, and invasion. Studies have shown dysregulation of miRNAs such as miR-15, miR-20a, miR-23a/b, miR-29c, miR-126, miR-142, miR-145, among others, influencing cell proliferation, invasion, and angiogenesis. For instance, miR-145 was found to be significantly upregulated in endometriosis. Additionally, let-7b's role in endometriosis might involve dysregulation of the p53 pathway and cell cycle control [14].

Carolina von Grothusen et al. highlighted dysregulated miRNAs in the endometrium of women with endometriosis, emphasizing their impact on endometriotic cell migration [15]. Ghasemi et al. identified altered expression of the miR-200 family in endometriosis patients, suggesting its diagnostic and therapeutic potential [16]. Ahmad Azam et al. discussed the potential of miRNAs as biomarkers for endometriosis diagnosis and their role in regulating apoptosis in endometriotic lesions [17]. Omeljaniuk et al. explored miRNAs as predictive factors for pregnancy diseases like miscarriage, proposing their use as minimally invasive diagnostic biomarkers [18]. Almiñana et al. (2025) identified 85 differentially expressed miRNAs in cumulus cells from endometriosis patients, linking these to oocyte quality, fertilization potential, and downstream embryo development. Key miRNA families (miR-17-5p/20a, miR-200, miR-145) are noted for their roles in endometrial remodeling and suppression of targets essential for trophoblast function [19]. Wang et al. (2024) demonstrated that reduced Syncytin-1, likely modulated by upstream miRNAs, impairs trophoblast invasion and epithelial-mesenchymal transition, underpinning pathology in abnormal pregnancies [20]. 

While the present study nominates miR-509-3p and miR-625-5p as novel candidates across multiple prediction platforms, these specific miRNAs have not been previously reported in direct experimental studies as regulators of Syncytin-1 or Syncytin-2. Earlier research primarily identified other miRNAs, such as the miR-200 family or miR-17-5p/20a, as key players in endometrial remodeling, trophoblast function, and endometriosis [21, 22]. 

The process of integrating data from diverse sources and prediction models allows for a comprehensive analysis that can lead to more robust and reliable results in identifying potential miRNAs involved in gene regulation. Collectively, aberrant expression of Syncytin-1 and Syncytin-2 can lead to various health implications, affecting placental development and pregnancy outcomes, and potentially contributing to conditions like preeclampsia and infertility. Understanding these proteins' molecular mechanisms and regulatory pathways is crucial for developing diagnostic markers and therapeutic interventions for associated health conditions.

This study identified miR-509-3p and miR-625-5p as potential regulators of Syncytin-1 and Syncytin-2, linked to endometriosis and miscarriage. These tools help in identifying differentially expressed miRNAs, understanding their roles in cellular processes, and potentially using them as biomarkers for disease diagnosis and prognosis. 

### Conflict of Interest:

The authors declare that they have no conflicts of interest.

### Authors’ Contribution:

SSM and MSH made significant contributions to the study’s conception and design. MGH and HS contributed to data collection, data analysis, and interpretation. SSM wrote the original manuscript, and all authors contributed significantly to its revision for important intellectual content. All authors approved the final version submitted for publication, and all authors accept responsibility for the statements made in the published article.

### Ethics approval and consent to participate:

Ethical approval was obtained from the Medical Ethics Committee of Tarbiat Modares University (IR.MODARES.REC.1401.251). 

### Data Availability:

The data sets generated or analyzed in the present study are available from the corresponding author upon reasonable request 

### Funding:

This work is based upon research funded by the Iran National Science Foundation (INSF) under project No.4016132.
