# Supplementary material for: Prediction of microRNAs affecting the syncytin-1 (HERV-W) and syncytin-2 (HERV-FRD) genes regulation in endometriosis and miscarriage
Source: Mol Biol Res Commun. 2026;15(1):63–70. doi: 10.22099/mbrc.2025.54457.2225 (PMC12673623; doi:10.22099/mbrc.2025.54457.2225)
Supplement: Supplementary file 1 — Table S1 [file mbrc-15-63-s001.pdf]

Molecular Biology Research Communications 2026;15(1):63-70 DOI:10.22099/mbrc.2025.54457.2225 MBRC
Original Article Open Access

Prediction of microRNAs affecting the syncytin-1 (HERV-W) and syncytin-2 (HERV-FRD) genes regulation in endometriosis and miscarriage

Mehdi Gholami-Barzoki[1], Somayeh Shatizadeh-Malekshahi[1,*], Mohammad Shayestehpour[2] Haleh Soltanghoraee[3]

1) Department of Virology, Faculty of Medical Sciences, Tarbiat Modares University, Tehran, Iran
2) Department of Bacteriology and Virology, Faculty of Medicine, Isfahan University of Medical Sciences, Isfahan, Iran
3) Reproductive Biotechnology Research Center, Avicenna Research Institute, ACECR, Tehran, Iran

Table 3a: List of predicted miRNA by DIANA targeting ERVW-1

Target Rank	Ensembl Gene Id	miRNA name	miTG score
1	ENSG00000242950 (ERVW-1)	hsa-miR-4764-5p	0.999991000663365
2	ENSG00000242950 (ERVW-1)	hsa-miR-302b-3p	0.999914086902561
3	ENSG00000242950 (ERVW-1)	hsa-miR-302e	0.999914086902561
4	ENSG00000242950 (ERVW-1)	hsa-miR-3934-5p	0.99991270776813
5	ENSG00000242950 (ERVW-1)	hsa-miR-302d-3p	0.999889128802559
6	ENSG00000242950 (ERVW-1)	hsa-miR-302c-3p	0.999886708172577
7	ENSG00000242950 (ERVW-1)	hsa-miR-302a-3p	0.999865780130068
8	ENSG00000242950 (ERVW-1)	hsa-miR-6895-3p	0.999829975996477
9	ENSG00000242950 (ERVW-1)	hsa-miR-5699-3p	0.999826792574528
10	ENSG00000242950 (ERVW-1)	hsa-miR-610	0.999824236114472
11	ENSG00000242950 (ERVW-1)	hsa-miR-4684-3p	0.999803875665664
12	ENSG00000242950 (ERVW-1)	hsa-miR-509-3p	0.999680713259469
13	ENSG00000242950 (ERVW-1)	hsa-miR-629-5p	0.999658371292083
14	ENSG00000242950 (ERVW-1)	hsa-miR-4667-3p	0.999376233304554
15	ENSG00000242950 (ERVW-1)	hsa-miR-7977	0.999325458335701

Table 3b: List of predicted miRNAs by DIANA targeting ERVFRD-1

Target Rank	Ensembl Gene Id	miRNA name	miTG score
1	ENSG00000244476 (ERVFRD-1)	hsa-miR-1303	0.999932543720518
2	ENSG00000244476 (ERVFRD-1)	hsa-miR-4687-3p	0.999787214672385
3	ENSG00000244476 (ERVFRD-1)	hsa-miR-625-5p	0.999728793069174
4	ENSG00000244476 (ERVFRD-1)	hsa-miR-876-3p	0.998219565879455
5	ENSG00000244476 (ERVFRD-1)	hsa-miR-6781-3p	0.995743191423699
6	ENSG00000244476 (ERVFRD-1)	hsa-miR-193a-5p	0.993645776945572
7	ENSG00000244476 (ERVFRD-1)	hsa-miR-6855-5p	0.992932932710868
8	ENSG00000244476 (ERVFRD-1)	hsa-miR-6165	0.992509783118052
9	ENSG00000244476 (ERVFRD-1)	hsa-miR-7154-5p	0.989742774747029
10	ENSG00000244476 (ERVFRD-1)	hsa-miR-3934-5p	0.983513477792745
11	ENSG00000244476 (ERVFRD-1)	hsa-miR-4728-5p	0.983253157610238
12	ENSG00000244476 (ERVFRD-1)	hsa-miR-5193	0.983134506466176
13	ENSG00000244476 (ERVFRD-1)	hsa-miR-2682-3p	0.977287247130125
14	ENSG00000244476 (ERVFRD-1)	hsa-miR-6780a-5p	0.97722343914932
15	ENSG00000244476 (ERVFRD-1)	hsa-miR-3622b-3p	0.977032092147944

Table 4a: A number of predicted miRNAs by miRWalk targeting ERVW-1

Target Rank	miRNA	Gene symbol	Score	Position	N pairing
1	**hsa-miR-6837-3p**	**ERVW-1**	**1.00**	**3UTR**	**18**
2	**hsa-miR-3619-3p**	**ERVW-1**	**1.00**	**3UTR**	**18**
3	**hsa-miR-6824-5p**	**ERVW-1**	**1.00**	**3UTR**	**18**
4	**hsa-miR-4651**	**ERVW-1**	**1.00**	**3UTR**	**18**
5	**hsa-miR-6753-3p**	**ERVW-1**	**1.00**	**3UTR**	**18**
6	**hsa-miR-3194-3p**	**ERVW-1**	**1.00**	**3UTR**	**18**
7	**hsa-miR-3174**	**ERVW-1**	**1.00**	**3UTR**	**18**
8	**hsa-miR-6735-3p**	**ERVW-1**	**1.00**	**3UTR**	**17**
9	**hsa-miR-6825-5p**	**ERVW-1**	**1.00**	**3UTR**	**17**
10	**hsa-miR-509-3p**	**ERVW-1**	**1.00**	**3UTR**	**17**
11	**hsa-miR-6843-3p**	**ERVW-1**	**1.00**	**3UTR**	**17**
12	**hsa-miR-5010-3p**	**ERVW-1**	**1.00**	**3UTR**	**17**
13	**hsa-miR-5088-3p**	**ERVW-1**	**1.00**	**3UTR**	**17**
14	**hsa-miR-365b-5p**	**ERVW-1**	**1.00**	**3UTR**	**17**
15	**hsa-miR-1185-1-3p**	**ERVW-1**	**1.00**	**3UTR**	**17**
16	**hsa-miR-92a-2-5p**	**ERVW-1**	**1.00**	**3UTR**	**16**

Table 4b: A number of predicted microRNAs by miRWalk targeting ERVFRD-1

Target Rank	miRNA	Gene symbol	Score	position	N pairing
1	**hsa-miR-6717-5p**	**ERVFRD-1**	**1.00**	**3UTR**	**17**
2	**hsa-miR-1909-5p**	**ERVFRD-1**	**1.00**	**3UTR**	**17**
3	**hsa-miR-3621**	**ERVFRD-1**	**1.00**	**3UTR**	**17**
4	**hsa-miR-564**	**ERVFRD-1**	**1.00**	**3UTR**	**17**
5	**hsa-miR-4731-3p**	**ERVFRD-1**	**1.00**	**3UTR**	**17**
6	**hsa-miR-6804-5p**	**ERVFRD-1**	**1.00**	**3UTR**	**17**
7	**hsa-miR-625-5p**	**ERVFRD-1**	**1.00**	**3UTR**	**17**
8	**hsa-miR-4259**	**ERVFRD-1**	**1.00**	**3UTR**	**17**
9	**hsa-miR-6732-5p**	**ERVFRD-1**	**1.00**	**3UTR**	**17**
10	**hsa-miR-4327**	**ERVFRD-1**	**1.00**	**3UTR**	**17**
11	**hsa-miR-6882-3p**	**ERVFRD-1**	**1.00**	**3UTR**	**17**
12	**hsa-miR-6090**	**ERVFRD-1**	**1.00**	**3UTR**	**16**
13	**hsa-miR-23a-5p**	**ERVFRD-1**	**1.00**	**3UTR**	**16**
14	**hsa-miR-105-3p**	**ERVFRD-1**	**1.00**	**3UTR**	**16**
15	**hsa-miR-149-3p**	**ERVFRD-1**	**1.00**	**3UTR**	**16**
16	**hsa-miR-323a-5p**	**ERVFRD-1**	**1.00**	**3UTR**	**16**
